# The future of AI clinicians: assessing the modern standard of chatbots and their approach to diagnostic uncertainty

**DOI:** 10.1186/s12909-024-06115-5

**Published:** 2024-10-11

**Authors:** Ryan S. Huang, Ali Benour, Joel Kemppainen, Fok-Han Leung

**Affiliations:** 1 Temerty Faculty of Medicine, University of Toronto, Health Centre at 80 Bond, St. Michael’s Hospital, 80 Bond Street, Toronto, ON M5B1X2 Canada; 2 Department of Medicine, Royal College of Surgeons in Ireland, Dublin, Leinster, Ireland; 3 Department of Family and Community Medicine, University of Toronto, Toronto, ON Canada

**Keywords:** Artificial intelligence, Diagnostic uncertainty, Decision making

## Abstract

**Background:**

Artificial intelligence (AI) chatbots have demonstrated proficiency in structured knowledge assessments; however, there is limited research on their performance in scenarios involving diagnostic uncertainty, which requires careful interpretation and complex decision-making. This study aims to evaluate the efficacy of AI chatbots, GPT-4o and Claude-3, in addressing medical scenarios characterized by diagnostic uncertainty relative to Family Medicine residents.

**Methods:**

Questions with diagnostic uncertainty were extracted from the Progress Tests administered by the Department of Family and Community Medicine at the University of Toronto between 2022 and 2023. Diagnostic uncertainty questions were defined as those presenting clinical scenarios where symptoms, clinical findings, and patient histories do not converge on a definitive diagnosis, necessitating nuanced diagnostic reasoning and differential diagnosis. These questions were administered to a cohort of 320 Family Medicine residents in their first (PGY-1) and second (PGY-2) postgraduate years and inputted into GPT-4o and Claude-3. Errors were categorized into statistical, information, and logical errors. Statistical analyses were conducted using a binomial generalized estimating equation model, paired t-tests, and chi-squared tests.

**Results:**

Compared to the residents, both chatbots scored lower on diagnostic uncertainty questions (*p* < 0.01). PGY-1 residents achieved a correctness rate of 61.1% (95% CI: 58.4–63.7), and PGY-2 residents achieved 63.3% (95% CI: 60.7–66.1). In contrast, Claude-3 correctly answered 57.7% (*n* = 52/90) of questions, and GPT-4o correctly answered 53.3% (*n* = 48/90). Claude-3 had a longer mean response time (24.0 s, 95% CI: 21.0-32.5 vs. 12.4 s, 95% CI: 9.3–15.3; *p* < 0.01) and produced longer answers (2001 characters, 95% CI: 1845–2212 vs. 1596 characters, 95% CI: 1395–1705; *p* < 0.01) compared to GPT-4o. Most errors by GPT-4o were logical errors (62.5%).

**Conclusions:**

While AI chatbots like GPT-4o and Claude-3 demonstrate potential in handling structured medical knowledge, their performance in scenarios involving diagnostic uncertainty remains suboptimal compared to human residents.

## Introduction

In recent years, the potential benefits of artificial intelligence (AI) in healthcare have been extensively explored [1, 2]. Among the barriers faced by outpatients at specialist care centers, more than half experience issues related to information availability and healthcare communication [3]. The advent of rapidly developing chatbots, such as ChatGPT, has highlighted the utility of AI in medical information dissemination and early patient education. These chatbots, with their advanced fluency and technical linguistic capabilities, offer the general patient population a wealth of easily accessible and accurate information [4–6]. They deliver context with careful consideration, potentially mitigating the occasionally alarming nature of highlighted internet search results [7, 8]. AI has already demonstrated benefits in triage, providing diagnostic results comparable to those of clinicians and offering safer recommendations on average [9, 10]. Furthermore, the rise of telemedicine as a medium for patient management presents an additional dimension suitable for language models [11].

Nonetheless, the intricacies of real-world medical practice go beyond static knowledge and involve domains fraught with diagnostic uncertainty. Diagnostic uncertainty arises when symptoms, clinical findings, and patient histories do not converge on a definitive diagnosis, necessitating nuanced interpretation, differential diagnosis, and often, iterative patient evaluation [12, 13]. This aspect of medical practice poses challenges even for seasoned clinicians, demanding a synthesis of experience, intuition, and continuous learning [14]. Previous studies have demonstrated that ChatGPT performs well on structured medical knowledge assessments, including the United States Medical Licensing Exam (USMLE) [15–19]. However, there is a paucity of research evaluating the performance of AI chatbots in scenarios involving diagnostic uncertainty.

In addition, it is crucial to consider the distinct ethical frameworks and training methodologies that different AI chatbots employ, as these factors can significantly influence their responses. For instance, ChatGPT is programmed with several moral principles, including privacy, non-maleficence, non-discrimination, and transparency, while Claude is trained within a virtue ethics framework, which emphasizes honesty and a context-sensitive approach [20–22]. This latter framework could potentially allow for more nuanced and empathetic responses, particularly in complex scenarios such as those involving diagnostic uncertainty. This study aims to assess the efficacy of AI chatbots in addressing medical scenarios characterized by diagnostic uncertainty and to compare the responses of chatbots trained on different ethical frameworks. Understanding the constraints and capabilities of AI chatbots in managing diagnostic uncertainty is crucial for their effective integration into clinical practice.

## Methods

### Study design

The Progress Test, conducted by the Department of Family and Community Medicine (DFCM) at the University of Toronto functions as a formative tool to evaluate the development of residents towards becoming Family Medicine Experts and supports their preparation for Board Certification. This biannual examination is structured as a closed, four-hour multiple-choice test, curated by subject matter experts in Family Medicine. Each item on the test presents four response options, labeled A through D. For this study, all questions from four Progress Tests administered between 2022 and 2023 to a cohort of 320 Family Medicine residents in their first (PGY-1) and second (PGY-2) postgraduate years that were tagged with the “diagnostic uncertainty” assessment objective, as highlighted by The College of Family Physicians in Canada, were extracted [23]. Diagnostic uncertainty questions were defined as those presenting clinical scenarios where symptoms, clinical findings, and patient histories do not converge on a definitive diagnosis, necessitating nuanced interpretation and differential diagnosis. The performance of medical residents (*N* = 320) on these questions was then compared against the performance of AI models GPT-4o and Claude-3 pro. Ethical approval for this study was granted by the University of Toronto Research Ethics Board.

### Data collection

To maintain study integrity, each question was input into both GPT-4o and Claude-3 in the same format as presented in the official examination, with multiple-choice answers labeled A through D, without any alterations or additional cues. Prior to entering each question, the chatbots’ conversation history was reset, and memory cleared to avoid any influence from previous interactions. The chatbots’ responses were reviewed by two independent reviewers (R.S.H., A.B.) to identify the chosen multiple-choice options. Each LLM was queried with the same question three times to assess for variability. Collected data included the date of question input, response length in characters, response time in seconds, the presence of a rationale for excluding other options, and the root cause of any incorrect responses. If the AI chatbot selected “all of the above” or “none of the above,” the answer was marked incorrect since these were not valid choices.

For each question, it was documented whether the response provided reasons for excluding incorrect options. Incorrect responses were classified into three mutually exclusive types by the reviewers (R.S.H., A.B.): statistical errors, information errors, and logical errors. Statistical errors were defined as mistakes in arithmetic calculations. Information errors occurred when the chatbot gathered incorrect information either from the question itself or external sources, resulting in an incorrect answer. Logical errors were identified when the AI chatbot had access to the correct information but failed to apply it accurately to arrive at the correct answer.

### Statistical analysis

The primary outcome of this study was to compare the performance of AI chatbots and PGY-1 and PGY-2 residents in answering questions involving diagnostic uncertainty. Secondary outcomes included comparing GPT-4o and Claude-3 performance, response length, response time, and the proportion of questions. Resident performance was calculated as an aggregate of the performance statistics on diagnostic uncertainty questions from the Family Medicine Progress Tests administered between 2022 and 2023, with 95% confidence intervals (CIs) derived using a binomial generalized estimating equation model. Chatbot performance was calculated based on the percentage of correct responses to the extracted questions. Analyses were stratified across each of the nine priority question areas. Paired t-tests were employed to compare means, and chi-squared tests were applied to compare proportions. We applied the Bonferroni correction method to control the family-wise error rate, ensuring that the significance level was appropriately maintained across the multiple comparisons [24]. A p-value threshold of 0.05 was set to determine statistical significance. Statistical analyses were conducted using Stata version 17.0 (StataCorp LLC, College Station, Texas).

## Results

**Fig. 1:**
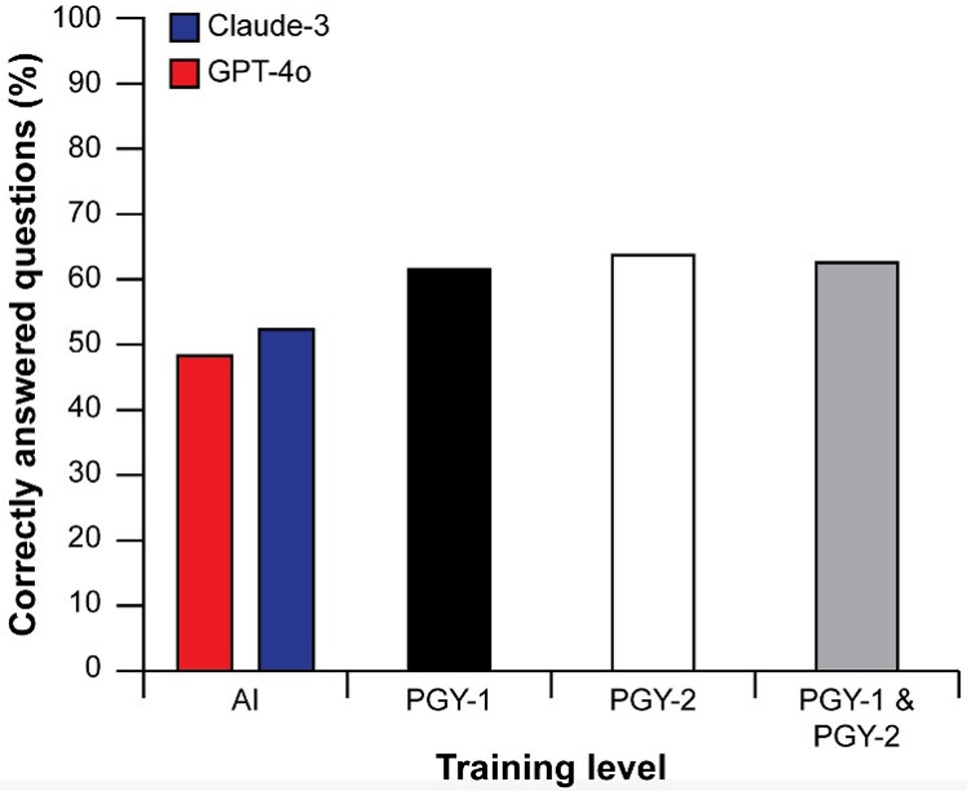
Resident and AI chatbot performance on diagnostic uncertainty questions

A total of ninety questions involving diagnostic uncertainty across nine categories within Family Medicine were included in the study selected from a total of 440 questions across four Progress Tests administered between 2022 and 2023 (Table 1). Overall, Claude-3 correctly answered 57.7% (*n* = 52/90) of the questions, while GPT-4o correctly answered 53.3% (*n* = 48/90) (Fig. 1). Both chatbots provided the same multiple-choice answer across all three trials for each question. The performance difference between the two chatbots was not statistically significant (*p* = 0.55). When comparing the performance of GPT-4o and Claude-3 to Family Medicine residents on diagnostic uncertainty questions, both chatbots underperformed relative to the residents. PGY-1 residents achieved an average correctness rate of 61.1% (95% CI: 58.4–63.7), and PGY-2 residents scored 63.3% (95% CI: 60.7–66.1), both significantly higher than the chatbots (*p* < 0.01). In specific categories, GPT-4o outperformed the residents in cardiovascular and gastrointestinal questions, with scores of 80% and 70%, respectively, compared to 64.5% and 65.7% among PGY-1 and PGY-2 residents (*p* < 0.01). Claude-3 excelled in geriatric care, mental health, and women’s health, scoring 70%, 80%, and 70%, respectively, outperforming the residents’ scores of 59.6%, 52.4%, and 56.4% (*p* < 0.01). Conversely, residents outperformed both chatbots in the endocrine, musculoskeletal, pediatric, and respiratory categories (*p* < 0.01).

**Table 1:** Comparison of GPT-4o and Claude-3 performance to Family Medicine residents on questions with diagnostic uncertainty

		GPT-4o (%)	Claude-3 (%)	PGY 1 Resident % Correct (*N* = 160)		PGY 2 Resident % Correct (*N* = 160)		PGY 1 + 2 Resident % Correct (*N* = 220)	
				%	95% CI	%	95% CI	%	95% CI
**Overall**		48 (53.3)	52 (57.7)	61.1	58.4–63.7	63.3	60.7–66.1	62.2	59.6–64.9
**Exam Category**									
	Cardiovascular	8 (80.0)	5 (50.0)	63.8	60.6–67.1	65.2	62.7–68.3	64.5	61.7–67.7
	Gastrointestinal	7 (70.0)	5 (50.0)	64.1	61.7–67.2	67.3	65.2–70.1	65.7	63.5–68.7
	Geriatric Care	4 (40.0)	7 (70.0)	58.7	55.4–61.2	60.4	58.5–63.5	59.6	57.0-62.4
	Endocrine	6 (60.0)	5 (50.0)	65.2	63.8–66.7	66.3	64.9–68.5	65.8	64.4–67.6
	Mental Health	3 (30.0)	8 (80.0)	51.3	48.5–54.8	53.4	50.7–56.8	52.4	49.6–55.8
	MSK	5 (50.0)	5 (50.0)	60.7	58.2–62.4	62.9	59.9–65.7	61.8	59.1–64.1
	Pediatric	5 (50.0)	5 (50.0)	65.9	63.2–68.4	68.6	65.4–71.2	67.3	64.3–69.8
	Respiratory	6 (60.0)	5 (50.0)	64.5	61.8–67.3	67.9	64.1–70.2	66.2	63.0-68.8
	Women’s Health	4 (40.0)	7 (70.0)	55.3	52.5–57.9	57.4	55.260.4	56.4	53.9–59.2

Claude-3 had a longer mean response time of 24.0 s (95% CI: 21.0-32.5) compared to GPT-4o, which had a mean response time of 12.4 s (95% CI: 9.3–15.3) (*p* < 0.01) (Table 2). In terms of response length, Claude-3 also produced longer answers, with a mean of 2001 characters (95% CI: 1845–2212) compared to GPT-4o’s 1596 characters (95% CI: 1395–1705) (*p* < 0.01). Both chatbots frequently provided rationales for other answer options, with Claude-3 doing so slightly more often than GPT-4o, although not statistically significant (86.7% vs. 78.9%; *p* = 0.17). Regarding the types of errors made, GPT-4o predominantly made logical errors, accounting for 62.5% of its mistakes, followed by information errors (18.8%) and statistical errors (18.8%). In contrast, Claude-3 had a lower proportion of logical errors at 44.7%, but higher rates of information errors (31.6%). An example of the output from GPT-4o and Claude-3 is provided in Table 3 and examples of errors are provided in Table 4.

**Table 2:** Comparison of response characteristics between GPT-4o and Claude-3

		GPT-4o	Claude-3	*P* Value
**Characteristics**				
	Response time (sec), mean (95% CI)	12.4 (9.3–15.3)	24.0 (21.0-32.5)	< 0.01
	Response length (characters), mean (95% CI)	1,596 (1,395.0–1,705.0)	2,001 (1,845-2,212)	< 0.01
	Rationale for other answer options, N (%)	71 (78.9)	78 (86.7)	0.17
**Reason for error**, **N (%)**		***n*** **= 48**	*n* **= 38**	
	Logical error	30 (62.5)	17 (44.7)	0.02
	Statistical error	9 (18.8)	6 (15.8)	0.72
	Information error	9 (18.8)	15 (31.6)	0.04

**Table 3:** Example GPT-4o and Claude-3 response to a diagnostic uncertainty question

Question	GPT-4o Response	Claude-3 Response
You are a family doctor working in a rural northern community of 3000 people. A 56-year-old female comes in today complaining of right neck pain extending into her right shoulder area. She is right hand dominant. Her symptoms started gradually over a year ago but have become more bothersome. She is now waking from sleep with neck discomfort and intermittent right arm tingling. The patient smokes approximately half a pack of cigarettes per day and drinks a bottle of beer every night with dinner. She has been struggling with a diet program for the past 5 years and over the last year, her BMI has increased from 34 to 36. On exam of the neck, she guards against pain but is able to flex her neck to half range and extend to one quarter range. Bilateral side flexion and rotation is reduced to half-range with ipsilateral stretching. Passive range of motion is full and pain free. Postural exam reveals a lordotic cervical spine and a kyphotic thoracic spine (she is slumping). Trigger points are positive over the affected area but not in the lower limbs. Neurologic exam is normal. What investigations should you consider? A. ESR, C-reactive protein, Rheumatoid Factor, ANA B. nerve conduction testing C. no immediate investigations required D. shoulder MRI **CORRECT ANSWER**: **C. no immediate investigations required**	B. nerve conduction testing For the 56-year-old female patient presenting with chronic right neck pain extending into her shoulder, along with intermittent tingling in her right arm, the following investigations should be considered: Option B: Nerve conduction testing - This test would be beneficial given the patient’s symptoms of tingling, which suggest a possible nerve involvement. Nerve conduction studies can help identify whether there is nerve damage or nerve dysfunction, which could be contributing to her symptoms. Option D: Shoulder MRI - An MRI of the shoulder might also be warranted due to the location of the pain and the duration of her symptoms. MRI can provide detailed images of the soft tissues, bones, and nerves in the shoulder area, helping to identify any structural causes of her pain, such as rotator cuff tears, bursitis, or other abnormalities that could be exacerbating her neck pain. Option A (ESR, C-reactive protein, Rheumatoid Factor, ANA) - These tests are generally used to investigate inflammatory or autoimmune conditions. While these could be considered if there was a suspicion of an inflammatory or autoimmune process, the clinical presentation described does not strongly suggest these as the primary cause of her symptoms. Option C (no immediate investigations required) - Given the chronicity and severity of the patient’s symptoms, particularly with the impact on her sleep and daily function, pursuing diagnostic investigations to identify the underlying cause is advisable rather than delaying further evaluation. Therefore, based on the clinical scenario, nerve conduction testing (B) would be the most relevant initial investigations to consider. This would help in assessing the possible neurological contributions to her symptoms, guiding further management.	C. no immediate investigations required Based on the clinical presentation and examination findings, the most appropriate investigations to consider for this patient would be C. No immediate investigations required. The patient’s history and physical examination findings are consistent with a diagnosis of cervical spondylosis or cervical osteoarthritis, which is a degenerative condition of the cervical spine that commonly occurs with aging. The gradual onset of neck pain radiating to the shoulder, intermittent arm tingling, and reduced range of motion with ipsilateral stretching are typical features of cervical spondylosis. The presence of trigger points in the affected area and the postural findings of a lordotic cervical spine and kyphotic thoracic spine (slumping posture) further support this diagnosis. In cases of uncomplicated cervical spondylosis, where the neurological examination is normal and there are no red flags (such as fever, significant trauma, or progressive neurological deficits), immediate investigations are generally not necessary. Option A, which includes inflammatory markers (ESR, CRP) and autoantibody tests (Rheumatoid Factor, ANA), would be more appropriate if there were concerns for an inflammatory or autoimmune condition, such as rheumatoid arthritis or other inflammatory arthropathies. Option B, nerve conduction testing, would be valuable if there were significant neurological deficits or suspicion of a peripheral neuropathy, but the normal neurological examination in this case does not warrant such testing at this stage. Option D, a shoulder MRI, might be considered if there were concerns for a rotator cuff or other shoulder joint pathology, but the clinical presentation and examination findings predominantly point towards a cervical spine etiology. In this case, the initial management would involve conservative measures, such as analgesics, lifestyle modifications (smoking cessation, weight loss, and postural correction), and physical therapy or exercise recommendations. If symptoms persist or worsen, or if neurological deficits develop, further investigations, such as cervical spine X-rays or an MRI, may be warranted at a later stage.

**Table 4:** Example of logical, statistical, and information errors

Logical Error	Statistical Error	Information Error
**Question**: You are a family physician working in a large, urban ER. A 3-year-old girl is brought in to your ER by her parents. The patient has been unwell for the past 5 days with cough, fever, runny nose, and sore throat. She has pink eyes bilaterally and a widespread maculopapular rash. For the past 24 h, she has become more lethargic and has not been able to drink any fluids. She has urinated once in the past 24 h. Her past medical history is unremarkable. Her developmental milestones have been up to date. However, she has not received any vaccinations - her parents are concerned about possible side effects of mercury. On examination, the patient is listless and lying on the stretcher. She is unresponsive to questions. Her blood pressure is stable, but she has an increased heart rate of 140 and a temperature of 39.5 °C rectally. You appreciate a full-body maculopapular rash as well as white lesions on the buccal mucosa. The cardiac and respiratory exam are normal. The abdomen is soft and non-tender. What is your most likely diagnosis? A. Disseminated varicella B. Kawasaki disease C. Measles encephalitis (CORRECT) D. Rubella **Chatbot Response**: B. Kawasaki disease. The patient’s presentation with fever, rash, conjunctivitis, and lethargy raises concerns for Kawasaki disease. Although the presence of white lesions on the buccal mucosa (Koplik spots) and the widespread maculopapular rash are suggestive of a viral infection, the combination of prolonged fever, rash, and mucosal involvement points to Kawasaki disease as the most likely diagnosis.” **Type of Error: Logical Error** **Explanation**: The chatbot fails to correctly apply clinical reasoning by prioritizing Kawasaki disease over measles encephalitis, despite the presence of key signs like Koplik spots and the patient’s unvaccinated status. Kawasaki disease typically presents with a more specific set of criteria, including extremity changes, lymphadenopathy, and mucous membrane changes. The chatbot had access to the correct information but failed to apply it accurately to arrive at the correct answer.	**Question**: You are a family physician working in a rural practice. A 68-year-old male presents to your office with poor pain control. His past medical history is notable for metastatic prostate cancer which has spread to bone. He is on dexamethasone 4 mg po daily. Six weeks ago, his opioid medications were morphine continuous release 20 mg po q12hours and morphine 5 mg q2h prn for breakthrough. This gave him good pain control for 3 weeks until he started needing more breakthrough medications. His medications were then increased to morphine continuous release 30 mg po q12hours and morphine 5 mg q2h prn for breakthrough. This has not resulted in good pain control. He has been on this regimen of medications for the past 3 weeks. His average daily total is 100 mg of morphine. The direct conversion to hydromorphone is 20 mg, and the direct conversion to fentanyl is 25mcg (patch). How should you manage this patient’s pain medication? A. Increase to daily total of morphine 120 mg B. Rotate to daily total of fentanyl 25mcg C. Rotate to daily total of hydromorphone 18 mg (CORRECT) D. Rotate to daily total of hydromorphone 24 mg **Chatbot Response**: D. Rotate to daily total of hydromorphone 24 mg. The patient’s current average daily morphine dose is 100 mg. When converting this to hydromorphone, the direct conversion suggests a dose of 20 mg hydromorphone. However, considering opioid rotation and the principle of cross-tolerance, I calculate that the equivalent hydromorphone dose should be 24 mg. Therefore, the appropriate management is to rotate to a daily total of hydromorphone 24 mg.” **Type of Error: Statistical Error** **Explanation**: The chatbot incorrectly calculates a total of 24 mg hydromorphone instead of the correct adjusted dose of 18 mg. This miscalculation leads to the recommendation of a higher-than-necessary hydromorphone dose, which could potentially result in overdose or increased side effects.	**Question**: You are a family physician working in a small suburban community doing clinic and house calls. A 70-year-old female was diagnosed with Parkinson’s disease 10 years ago and was placed on levodopa-carbidopa. Over the past 2 months, her caregiver has noticed fluctuating cognition and alertness as well as possible visual hallucinations. A recent home visit and physical exam reveals normal vitals. She has a shuffling gait and resting tremor. You perform an MMSE which scores 12/30.Soon after the visit, the patient is admitted to the hospital after fracturing her hip. She presents with delirium. She is brought in by her caregiver (who is her power of attorney for personal care). The caregiver provides consent for surgery. The patient’s ex-spouse disagrees, as does her estranged son. How should you proceed? A. Contact hospital consent and capacity board B. Follow care directions from caregiver (CORRECT) C. Follow care directions from ex-spouse D. Follow care directions from son **Chatbot Response**: D. Follow care directions from son. The patient’s estranged son has raised concerns about the surgery and is the next of kin and holds the power of attorney, making him the appropriate person to make medical decisions on behalf of the patient. Therefore, you should follow the care directions from the son.” **Type of Error: Information Error** **Explanation**: The chatbot incorrectly states that the son holds the power of attorney, when the prompt stated the caregiver holds the power of attorney for personal care, which legally grants the caregiver the authority to make medical decisions on behalf of the patient. Gathering the wrong information here leads to an incorrect recommendation.

## Discussion

Our study compared the performance of AI chatbots, GPT-4o and Claude-3, against Family Medicine residents in addressing diagnostic uncertainty using questions from official progress examinations at the University of Toronto DFCM. Overall, both chatbots underperformed relative to the residents. Although Claude-3 generated longer and more rationale-rich responses, it was more prone to information errors compared to GPT-4o.

In a previous study examining chatbot performance on a Family Medicine Progress Test, ChatGPT demonstrated superior performance compared to the best-performing resident, highlighting its capability in handling well-defined medical knowledge assessments [16]. However, the results from our novel study, focusing solely on questions involving diagnostic uncertainty, reveal a significant shift in performance dynamics. Both GPT-4o and Claude-3 performed worse than first-year Family Medicine residents. This discrepancy underscores the heightened complexity and nuanced judgment required in scenarios characterized by diagnostic uncertainty, which current AI systems struggle to navigate effectively [25].

There are several plausible explanations for why AI systems struggle with this dimension of healthcare provision. Primarily, AI systems lack the contextual understanding required to appreciate the intricacies of modern medicine [26]. Their algorithms, trained on statistical patterns within limited data sets, are ill-suited to handle rare disease presentations, compounding illnesses, and conflicting clinical data [26, 27]. This bias towards trained data leads AI systems to fill gaps in information with assumptions, resulting in incomplete and incorrect diagnoses [28]. For instance, AI systems like GPT-4o have been found to prefer clinical diagnoses over pathological causes, such as selecting frontotemporal dementia over frontotemporal lobar degeneration, possibly influenced by the available training data [29]. The authenticity and quality of the training data used by these systems are of great consequence [30, 31]. The validity, diversity, and representativeness of the datasets included reinforce the decision-making capacity of the system when approaching rare and complex cases. Conversely, human physicians possess a wealth of experience regarding disease presentation, allowing them to consider individual circumstances, history, prevalence, and additional investigations to make a holistic diagnostic process [32]. This level of nuanced understanding is challenging to encode into an AI system.

Another critical consideration is that ChatGPT has been found incapable of recognizing and expressing uncertainty [33]. A cornerstone of modern medical practice and the training of medical practitioners is the risk assessment process, which involves calculating the probabilities of failure or complications while considering the patient’s comorbidities and leveraging these against the potential benefits of the intervention [33]. Salihu et al. (2024) describe seven cases where AI selected invasive treatments, whereas human physicians determined that medication would suffice [34]. These decisions were based on a complex array of considerations involving frailty, comorbidities, and life expectancy [34]. A similar finding emerged in our study, with ChatGPT recommending investigations when none were required. AI systems tend to answer decisively and confidently, often overestimating their confidence level regardless of the validity of their responses. ChatGPT was also found to be incapable of using low confidence levels to increase the number of unanswered questions in a sample exam designed to challenge its strategic capabilities [33]. This overconfidence may be considered a linguistic trait essential to the marketability of the system, but it underscores a significant concern regarding its integration into healthcare delivery.

The observed performance differences between GPT-4o and Claude-3 in specific medical domains can potentially be attributed to the distinct ethical frameworks and training methodologies employed for each AI system. GPT-4o performed better in areas such as cardiovascular and gastrointestinal health, possibly due to its programming with a predetermined set of moral principles, including privacy, non-maleficence, non-discrimination, and transparency [21]. These principles may guide GPT-4o towards clear, decisive answers in well-defined medical scenarios where established protocols and concrete data are available, as is often the case in cardiovascular and gastrointestinal health. Conversely, Claude excelled in mental health, women’s care, and geriatric care, which may be attributed to its training based on virtue ethics, emphasizing honesty and intention within a flexible, context-sensitive framework [20]. The nuanced and individualized nature of these domains likely benefits from the virtue ethics approach, which allows for more empathetic and contextually appropriate responses. Mental health, women’s care, and geriatric care often involve complex, subjective factors and require a deep understanding of the patient’s unique circumstances. Claude’s ethical framework may better equip it to navigate these complexities, providing more thoughtful and tailored responses. Consistent with the literature, the majority of ChatGPT’s errors were also in logical reasoning [16]. Given that diagnostic uncertainty questions often arise from incomplete or highly nuanced information that escapes common medical databases, ChatGPT may simply overlook steps in logical reasoning [35]. Claude-3, in contrast, committed fewer logical errors. These findings suggest that the ethical training heuristics embedded in AI systems may influence their performance across different medical domains, especially in scenarios involving diagnostic uncertainty.

In addition to comparing the accuracy of each chatbot, Claude-3 responded to the prompts more slowly than ChatGPT, but its answers were generally longer on average. Longer response times may suggest that the LLMs are engaging in more detailed analysis, which could correlate with higher accuracy in scenarios requiring nuanced decision-making. This is partially supported by our findings where Claude-3, with longer response times, performed slightly better than GPT-4o, although the difference was not statistically significant. However, it is important to recognize that response times are also subject to server latency and other external factors, which could introduce variability unrelated to the LLM’s cognitive processing. Therefore, while response time provides some insight into the LLM’s functioning, its interpretation should be approached with caution.

Our investigation is subject to several limitations. Given that ChatGPT is updated regularly, incorporating user feedback, its responses to identical queries might vary over time. We attempted to control for these variations by having the models respond to all multiple-choice questions on the same day, and we confirmed the consistency of responses across two different web browsers and three trials per question. It is essential to consider that the findings of this study are relevant to the specific period when they were collected, as the capabilities of both GPT-4o and Claude-3 are expected to evolve. Moreover, these models depend on cookies for optimal functionality and their responses can be affected by prior inputs. To counteract this, we regularly cleared conversation histories and memory before entering new prompts. Another consideration is that our questions were multiple-choice; the models’ performance might differ with open-ended questions or tasks requiring prioritization.

## Conclusions

In conclusion, while AI chatbots like GPT-4o and Claude-3 show promise in handling structured medical knowledge, their performance in scenarios involving diagnostic uncertainty remains suboptimal compared to human residents. The influence of ethical rule sets on AI performance warrants further investigation, as a virtue ethics framework may offer some advantages in managing complex clinical decisions. Future studies should focus on exploring the capabilities of AI in authentic healthcare contexts, particularly in its role as a clinical decision support tool intended to augment, not replace, physician clinical reasoning.

## Data Availability

The data that support the findings of this study may be requested at ry.huang@mail.utoronto.ca with support from the principal investigator Fok-Han Leung.

## Declarations

### Clinical trial number

N/A. This study is not a clinical trial.

## Abbreviations

AI: Artificial Intelligence
PGY-1: Postgraduate Year 1
PGY-2: Postgraduate Year 2
DFCM: Department of Family and Community Medicine
CI: Confidence Interval
USMLE: United States Medical Licensing Exam
LLM: Large Language Model
